# 

**Published:** 1972-01

**Authors:** 


The
Bristol Medico-Chirurgical Journal
(Incorporating the Medical Journal of the South-West)
Established 1883

Vol. 87 (i) | JANUARY 1972 | No. 321

### BRISTOL MEDICO-CHIRURGICAL JOURNAL

**Vol. 87**
**1972**

Editor: N. J. Brown, M.B., F.R.C.P., F.R.C.Path.

Published Quarterly

Annual Subscription £2 Post Free

### BRISTOL MEDICO-CHIRURGICAL SOCIETY

**President 1971-72 : Dr. C. C. Morgans**

**President-elect : Dr. James Macrae.**

**Honorary Secretary : Dr. I. S. Bailey, 7 Percival Road, Bristol 8.**

**Honorary Treasurer : Dr. Frank Ross, Bristol Royal Infirmary, Bristol 2.**

The Society was founded in 1874. In 1883 publication of the Journal began and has continued quarterly ever since then. During the years 1949 to 1962 it appeared under the title of "The Medical Journal of the South West".

### THE BRISTOL MEDICO-CHIRURGICAL JOURNAL

**Editorial Committee**

Editor Dr. N. J. Brown, Southmead Hospital, Bristol

Members Dr. Rhys Davies.
Dr. M. B. Lennard.
Professor R. C. Wofinden.
Dr. D. W. Wright.
The Hon. Treasurer and Hon. Secretary of the Society.

Business Manager Dr. P. J. F. Baskett, Frenchay Hospital, Bristol.

Secretary Mr. B. P. Jones, The Library, Medical School, University Walk, Bristol BS8 1TD.

### NOTICE TO CONTRIBUTORS

The Journal is especially concerned to provide a place for the recording of medical thought, interests, and practice in and around Bristol. It therefore welcomes articles that, as well as being of immediate interest to members, will document the local and contemporary medical scene for our successors.

Original articles are invited provided that they have not been published elsewhere. References in the text should be by author's name, with year of publication in parenthesis; they should be listed at the end in alphabetical order, the name of each journal or book being given in full—not abbreviated—and the title of the article added. Illustrations should be supplied ready for reproduction. Line drawing should be in Indian ink on firm white paper or board. The author will be informed if it is found necessary to ask him to pay for the making of blocks.

The following contributions will be especially welcome; notes of forthcoming events, meetings held, degrees conferred, staff appointments, honours and public appointments and other local news.

Twenty-five reprints of his article will be supplied free to each contributor. Quotations for larger quantities will be sent out with proofs and must be ordered when the corrected proofs are returned.

### The Medical Library

In 1890 the Bristol Medico-Chirurgical Society founded a medical library which in 1892 was combined with that of the Bristol Medical School. This became the University of Bristol Medical Library in 1925 and an agreement in that year confirmed the privilege of Members of the Society to use the University Medical Library.

### A Selection of Recent Additions to the Medical Library

ABRAMSON. H. ed.: Symposium on the functional physiopathology of the fetus and neonate: clinical correlations. (Mosby)

ALLEGRA, G. et al.: Gastroscopy with the fiberscope. (Piccin Medical Books)

ARENA, J. M.: Poisoning: toxicology, symptoms, treatments. Ed 2. (Thomas)

BASERGA, R. ed.: The cell cycle and cancer (Biochemistry of disease, 1.) (Dekker)

BENDALL, J. R.: Muscles, molecules and movement. (Heinemann)

BEVAN, J. A. et al.: Physiology and pharmacology of vascular neuroeffector systems. (Karger)

BICKEL, H. et al., eds.: Phenylketonuria. (Thieme)

BIRDWOOD, G. F. B. et al., eds.: Parkinson's disease: a new approach to treatment. (Acad. Press)

BLOODWORTH, J. M. B. ed.: Endocrine pathology. (Williams & Wilkins)

BLUM, J. J. ed.: Biogenic amines as physiological regulators (Prentice-Hall)

BOYCOTT, J A: Natural history of infectious disease. (Arnold)

BRECK, L. W.: An atlas of the osteochondroses. (Thomas)

BUCHER, N. L. R. & MALT, R. A.: Regeneration of liver and kidney (Little, Brown)

BURNET, M.: Genes, dreams and realities. (Medical and Technical Publ. Co.)

BURTON, A. L.: Cinematographic techniques in biology and medicine. (Acad. Press)

CIBA FOUNDATION: High altitude physiology. (Churchill)

CIBA FOUNDATION: Strategy of the viral genome. (Churchill)

COCKETT, R.: Drug abuse and personality in young offenders. (Butterworth)

COLOMBO, J. P.: Congenital disorders of the urea cycle and ammonia detoxication (Karger)

COWIE, A. T. & TINDAL, J. S.: Physiology of lactation. (Arnold)

CRUZ-COKE, R.: Color blindness: an evolutionary approach. (Thomas)

DAVIDSON, C. S.: Liver pathophysiology: its relevance to human disease. (Churchill)

DISBREY, B. D. & RACK, J. H.: Histological laboratory methods. (Churchill Livingstone)

DOLL, R. et al., eds.: Cancer incidence in five continents. Vol 2—1970. (Springer for International Union against Cancer)

DOUGLAS, D. M.: Surgical departments in hospitals—the surgeon's view. (Butterworth)

DUNLOP, R. H. & MOON, H. W. eds.: Resistance to infectious disease. (Saskatchewan Univ. Press)

EDWARDS, N. A. & HASSALL, K.: Cellular biochemistry and physiology. (McGraw-Hill)

ERANKÖ, O. ed.: Histochemistry of nervous transmission. (Elsevier)

FALCONER, I. A. ed.: Lactation. (Butterworth)

FELDMAN, S. A. & CRAWLEY, B. E. ed.: Tracheostomy and artificial ventilation. Ed 2. (Arnold)

FISHER, J. W.: Renal pharmacology. (Butterworth)

FISHMAN, A. P. & HECHT, H. H. eds.: The pulmonary circulation and interstitial space. (Chicago Univ. Press)

FORSCHER, B. K. & HOUCK, J. C. eds.: Immunopathology of inflammation. (Excerpta Medica)

FUCHS, F. & KLOPPER, A. eds.: Endocrinology of pregnancy. (Harper & Row)

GANONG, W. F. & MARTINI, L. eds.: Frontiers in neuroendocrinology, 1971. (Oxford Univ. Press)

GAUNT, W. A.: Microreconstruction. (Pitman)

GIBBS, M. ed.: Structure and function of chloroplasts. (Springer)

GILSTON, A. & RESNEKOV, L.: Cardio-respiratory resuscitation. (Heinemann)

GORDON, B. L. & FORD, D. K.: Essentials of immunology. (Davis)

GOURLEY, D. R. H.: Interactions of drugs with cells. (Thomas)

GRESHAM, G. A.: A colour atlas of general pathology. (Wolfe) (Presented by the Association of Applied Biologists)

GUYTON, A. C.: Basic human physiology: normal function and mechanisms of disease. (Saunders)

HALL, R. H.: The modified nucleosides in nucleic acids. (Columbia Univ. Press)

HAMBURGH, M. & BARRINGTON, E. J. W. eds.: Hormones in development. (Appleton-Century-Crofts)

HARRIS, J. E. ed.: Proceedings of the 5th Leukocyte Culture Conference. (Acad. Press)

HEILBRONN, E. & WINTER, A. eds.: Drugs and cholinergic mechanisms in the central nervous system. (Almqvist & Wiksell)

HUCKSTEP, R. L.: A simple guide to trauma. (Livingstone)

JAMIESON, K. G.: A first notebook of head injury. Ed 2. (Butterworth)

JAYSON, M. ed.: Total hip replacement. (Sector Publishing Ltd.)

JENNINGS, R. K. & ACKER, R. F.: The protistan kingdom: protists and viruses. (Van Nostrand)

JORGENSEN, C. B.: John Hunter, A. A. Berthold and the origins of endocrinology. (Odense Universitetsforlaget)

JONES, R. S. & OWEN-THOMAS, J. B.: Care of the critically ill child. (Arnold)

JOPLING, W. H.: Handbook of leprosy. (Heinemann)

JOSLIN, C. A. F. & GLEAVE, E. N. eds.: Clinical management of advanced breast cancer (2nd Tenovus Workshop) (Alpha Omega Alpha)

KING, R. et al.: Patterns of residential care. (Routledge)

LAJTHA, A. ed.: Alterations of chemical equilibrium in the nervous system (Handbook of neurochemistry, 6) (Plenum Press)

LAKI, K. ed.: Contractile proteins and muscle. (Dekker)

LANE-PETTER, W. & PEARSON, A. E. G.: The laboratory animal—principles and practice. (Acad. Press)

LAURENT, J. et al.: Hypoglycaemic tumours. (Excerpta Medica)

LIVINGSTON, R. B. & CARTER, S. K.: Single agents in cancer chemotherapy. (Plenum Press)

McCAMMON, R. W.: Human growth and development. (Thomas)

McCOMISH, P. B. & BODLEY, P. O.: Anaesthesia for neurological surgery. (Lloyd-Luke)

McLACHLAN, G. & McKEOWN, T. eds.: Medical history and medical care. (Oxford Univ. Press for Nuffield Prov. Hosp. Trust)

MALTRY, E. ed.: Benign and malignant tumors of the urinary bladder. (Lewis)

MARAMOROSCH, K. & KURSTAK, E. ed.: Comparative virology. (Acad. Press)

MATHÉ, G. et al.: Bone marrow transplantation and leucocyte transfusions. (Thomas)

MATTHEWS, W. B. & MILLER, H.: Diseases of the nervous system. (Blackwell)

MEYER, A.: Historical aspects of cerebral anatomy. (Oxford Univ. Press)

MIHICH, E.: Drugs and cell regulation: organizational and pharmacological aspects on the molecular level. (Acad. Press)

MOORE, G. P.: Organic voice disorders. (Prentice-Hall)

NEURATH, A. R. & RUBIN, B. A.: Viral structural components as immunogens of prophylactic value (Karger)

NEWSAM, J. E. & PETRIE, J. J. B.: Urology and renal medicine. (Livingstone)

NOWINSKI, W. W. & GOSS, R. J.: Compensatory renal hypertrophy. (Acad. Press)

OHLOFF, G. & THOMAS, A. F. eds.: Gustation and olfaction. (Acad. Press)

PAOLETTI, R. & DAVISON, A. N. eds.: Chemistry and brain development. (Plenum Press)

PEASE, D. C.: Cellular aspects of neural growth and differentiation. (California Univ. Press)

PHILLIPS, D. M. P. ed.: Histones and nucleohistones. (Plenum Press)

POLIAKOV, G. I.: On the neuronal organization of the brain. (Mir Publishers)

REGAMEY, R. H. et al., eds.: International symposium on enterobacterial vaccines. (Karger)

REVILLARD, J. P. ed.: Cell-mediated immunity: *in vitro* correlates. (Karger)

ROBISON, G. A. et al.: Cyclic AMP. (Acad. Press)

ROITT, I. M.: Essential immunology. (Blackwell)

RUSSELL, W. R.: The traumatic amnesias. (Oxford Univ. Press)

SCHONFIELD, H. & DE WECK, A. eds.: Mode of action (Antibiotics and chemotherapy, 17) (Karger)

SHEARN, M. A.: Sjögren's syndrome. (Saunders)

SHERWOOD, N. M. & TIMIRAS, P. S.: A stereotaxic atlas of the developing rat brain. (California Univ. Press)

SILVESTRI, L. G. ed.: Ribonucleic acid polymerase and transcription (1st Lepetit Colloquium) (North-Holland)

SIMPSON, L. L.: Neuropoisons: their pathophysiological actions. Vol 1: Poisons of animal origin. (Plenum Press)

SMITH, M. & WILLIAMS, R. eds.: Immunology of the liver. (Heinemann)

SMITH, R. T. & LANDY, M. eds.: Immune surveillance. (Acad. Press)

SNAPPER, J. & KAHN, A.: Myelomatosis. (Karger)

SOPHIAN, J.: Pregnancy nephropathy. (Butterworth)

STANDING MEDICAL ADVISORY COMMITTEE: The organisation of group practice. (HMSO)

STERLING, T. D. et al.: Visual prosthesis. (Acad. Press)

SUNDERMAN, F. W. & SUNDERMAN, F. W.: Laboratory diagnosis of liver diseases. (Hilger)

TAYLOR, J. L.: The doctor and negligence. (Pitman) Presented by Medical Protection Soc. Ltd.

THAL, A. P. et al.: Shock: a physiologic basis for treatment. (Year Book Med. Publ.)

TRIGGLE, D. J. et al., eds.: Cholinergic ligand interactions. (Acad. Press)

WALKER, W. F. & JOHNSTON, I. D. A.: The metabolic basis of surgical care. (Heinemann)

WEISS, D. W. ed.: Immunological parameters of host-tumor relationships. (Acad. Press)

WENDER, P. H.: Minimal brain dysfunction in children. (Wiley)

WYSS, O. & EKLUND, C. E.: Micro-organisms and man. (Wiley)

ZINN, W. M.: Idiopathic ischemic necrosis of the femoral head in adults. (Thieme)

ZÜLCH, K. J. ed.: Cerebral circulation and stroke. (Springer)

### Society Reports

### BRISTOL MEDICO-CHIRURGICAL SOCIETY

The Annual General Meeting was held in the Medical School on 13th October 1971. Dr. C. R. Tribe showed pathological specimens, Dr. G. R. Airth demonstrated skiagrams and Mr. B. P. Jones arranged a display of medical books. Dr. Beryl Corner resigned the chair to the new president Dr. C. C. Morgans, who delivered his presidential address which is to be published in a later number of the Journal. Dr. James Macrae was declared President-Elect and other officers elected. Reports were presented from the Hon. Secretary and Hon. Treasurer. The Hon. Editor of the Journal reported as follows:—

"Publication of the Journal has been somewhat hindered in the past year by lack of funds. Printing costs have risen considerably in recent years and it became clear that the contribution from members' subscriptions toward the Journal and the price charged to subscribers had both become quite unrealistic. It was very gratifying therefore that earlier this year the Society agreed to an increase in subscription so that the Journal could continue. The money needed was not, nevertheless, immediately forthcoming and, to avoid running into debt, it was decided to slow down publication until the bigger income became available in the autumn.

"The January issue—very much an "economy number", almost devoid of illustrations—was published late. The April number has just appeared and the July and October numbers will, we hope, be produced in quick succession before the end of the year, so that normal publication can be resumed in 1972. It might be asked why we did not produce one composite number to complete the present volume, but to have done so would have been unfair to our advertisers who pay for, and naturally expect, their advertisements to appear four times in the year. It would also be unfair to the suppliers of exchange journals for the library and Mr. Jones, the librarian, reports that the non-appearance of the journal since January has been noticed and that he has received enquiries from all sides.

"It is most encouraging to report that there is no lack of material for publication and a steady stream of interesting and informative articles has come in throughout the year. We are most grateful to all those Members who have written articles and trust that their example will continue to be followed by others. The Society's journal, believe it or not, is read throughout the world, but its quality and success depend, in the long run, on the enthusiasm and industry of the Society's members themselves.

"I should like to thank the members of the Editorial Committee for their invaluable help and advice during the year ,and, once again, the Society for its generous financial support."

On 10th November 1972 the Clinical Meeting was held at Stoke Park Hospital and on 8th December 1971 Dr. P. S. Byrne addressed the Society on "The Hard Times of General Practice". Dr. O. C. Lloyd showed pathological specimens and Dr. Rhys Davies demonstrated skiagrams. Mr. Jones again arranged a display of medical books.

On 24th November at a joint meeting with Galenicals Mr. P. R. Steele gave a fascinating account of his experiences on the recent Everest expedition in a talk entitled "Doctor on Everest."

### SOUTH-WEST ORTHOPAEDIC CLUB

A Meeting of the Club was held in Bath on the 6th November, 1971.

**Fourth Kenneth Pridie Memorial Lecture**—The Fourth Kenneth Pridie Memorial Lecture was delivered at Bath on the 6th November 1971 by Professor Erik Moberg (Gotherberg). He chose for his subject "Differentiation of Sensibility as a Basis for Orthopaedic and Hand Surgery". Professor Moberg first paid tribute to Kenneth Pridie's energy, practical ability and originality of thought. He hoped to handle his subject with the same broadness of concept and simplicity he had found in Pridie's writings.

The use of the simple traditional methods of the cotton-wool wisp and pin-prick were of little value in assessing sensory function and, in fact, could be quite misleading. It was possible if the responses to these methods of testing the modalities of touch and pain were normal to come to the quite erroneous conclusion that the sensibility being tested was also normal. A short film demonstrated the point in question, illustrating the reduced function present in a hand with a median herve lesion, where partial recovery was sufficient to give normal response to testing with cotton-wool and pin. The patient with impaired sensory function in the hand will always move the grip to the area with the best sensory function, even if that area's motor function is impaired.

In a search for improved methods of testing sensory function the two point discrimination test had proved to be the most useful. It should be performed with a blunt pointed instrument, and here a simple method using a paper clip was recommended and demonstrated by means of a second short film.

The value of the two point discrimination test was illustrated by its ability to detect reduced sensibility after the failure of the more traditional tests in the limbs of three patients with trophic ulcer problems resulting from congenital and traumatic lesions of the peripheral nervous system.

Lack of protective function was a factor in some cases of diabetic gangrene and impairment of two point discrimination could always be demonstrated in such cases, even when the response to cotton-wool and pin-prick was normal.

In children where the two point test cannot be used, it had been found valuable to allow the child to pick up a piece of coloured chalk, thus visually demonstrating the areas of the hand used. Again it could be seen that grip followed the sensory rather than the motor function. Skin areas used in gripping were very small. Appreciation of this had led to the development of the neuro-vascular island flap. Professor Moberg demonstrated the first use by him of such a flap in a hand injury eighteen years earlier.

Next the problem of proprioception and the siting of the peripheral receptors was discussed. Are they in muscle, tendon, joint or skin? Modern physiology textbooks come down in favour of the joints but he felt the skin receptors have an important role. Limiting the problem to the study of the conscious sensation of position and passive motion, he had by studying patients with neuro-vascular island flaps to otherwise insensitive fingers and a patient with a totally destroyed interphalangeal joint which had been replaced by a silastic joint, been able to isolate joint sensation from skin sensation. In these cases where there could be no nerve supply to the joints, the patient still had conscious appreciation of position and passive motion. He could only conclude from these experiences that the cutaneous receptors play an important role in the sensation of position and movement.

**Ankle Fractures in Association with Fractures of the Tibial Shaft**—Mr. H. K. Lucas (Bristol) reported that ankle fractures in association with tibial shaft fractures are scarcely mentioned in the literature.

In a consecutive series of 400 closed tibial shaft fractures treated in four years there were 200 compression plating operations in which no plaster casts have been used and in the cast treated cases there has always been a delay of two to ten days before plaster was applied. In consequence pre- and post-operative X-rays of the ankle as well as the shaft are available for study. Analysis of this series has shown:—

(a) Seventy-five per cent tibial shaft fractures are transverse oblique and twenty-five per cent spiral.
(b) One third of spiral shaft fractures have a concomitant fracture in the ankle joint sometimes by downward extension of the spiral in the tibia, but the majority are independent fractures in order of frequency in the lateral malleolus, posterior malleolus, medial malleolus, and anterior malleolus, and occasionally two or three malleoli together and in one case an ankle diastasis in association with a posterior malleolar fracture.

In only two patients out of three hundred with a transverse oblique fracture was a concomitant ankle fracture identified.

Of the twenty-three spiral shaft fractures with additional ankle fracture the shaft was treated by internal fixation in twelve and by cast fixation in eleven. At a one year follow-up ankle joint stiffness measured on a Hicks type goniometer averaged five degrees in the cases with internal fixation and twenty-two degrees in the cast treated cases.

There is a case for delaying the definitive treatment of a shaft fracture by the early use of a heel wire and traction, to give time to identify the cases — mainly spirals — with ankle damage, to permit consideration of internal fixation by plating or nailing for early ankle mobilisation with or without malleolar screw fixation depending on the stability of the joint.

**Anterior Synovectomy of the Rheumatoid Knee for Posterior Cysta**—Dr. M. Jayson (Bristol and Bath) reported that quadriceps contraction normally produces a negative pressure within the knee joint but in the presence of rheumatoid disease and effusions positive pressures are developed. It has been shown (Jayson and Dixon, 1970) that popliteal cysts associated with rheumatoid joints are linked with the joint by a valvular connection allowing fluid to pass from the knee to the joint but not in the reverse direction. This reduces the volume of effusion within the knee and therefore limits the pressure increase developed during joint use. Treatment of these cysts should therefore logically be directed primarily at the knee rather than at the cyst. A series of nine rheumatoid knees associated with such cysts were described. They were treated by anterior synovectomy with no attempt being made to remove the cyst. Changes in the cyst size were followed by clinical examination, arthrography and manometry. In all cases the cysts diminished in size and pressure, and in six they completely disappeared. In all cases they ceased to be a clinical problem. Anterior synovectomy of the rheumatoid knee is the correct procedure for treating posterior cysts.

**Deformities of the Thumb in Rheumatoid Arthritis** — Mr. A. H. C. Ratliff (Bristol) reported the results of a study of the hands of 33 patients suffering from rheumatoid arthritis. 40 of the 66 hands demonstrated a deformity of the thumb, the deformity being the intrinsic plus or Z deformity in 20 hands, the unstable thumb in 14 hands, the adducted thumb in 22 hands, and other deformities from rupture of the extensor pollicis longus or flexor pollicis longus in 7 hands.

Though deformity was present in 40 thumbs, symptoms such as pain or loss of function were present in only 15 thumbs.

In considering treatment it is important to appreciate that stability is more important than movement in the thumb, especially at the metacarpal-phalangeal and interphalangeal joints. The most common operation for patients with deformed thumbs is an arthrodesis of one or other of these two joints, the aim being to relieve pain and restore stability. Other operations are only rarely indicated.

### WEST COUNTRY RHEUMATISM CLUB

The inaugural meeting of the West Country Rheumatism Club was held in the Royal National Hospital for Rheumatic Diseases in Bath on Wednesday the 17th November, 1971. This club has been formed so that those doctors interested in rheumatic diseases in the region can meet about twice a year. The following officers were elected:

Hon. Chairman: Dr. M. I. V. Jayson.
Hon. Secretary: Dr. R. K. Jacoby.
Hon. Treasurer: Dr. A. K. Thould.

The programme of the first meeting included a talk by Dr. G. D. Kersley tracing the history of rheumatology in the region. There were then two case presentations. Dr. A. Kahn showed a case of ankylosing spondylitis without sacroiliitis and Dr. R. K. Jacoby showed a case of gout associated with hypokalaemic alkalosis.

Six ten minute papers were delivered and these included:—

Dr. A. St. J. Dixon: Trouble with Feet.
Dr. J. Cosh—Rheumatoid heart valve disease.
Dr. M. Jayson: Current research in intervertebral discs.
Dr. Joan Davies: Cost/benefit analysis of hip arthroplasty.
Dr. A. Collins: Steroids and lysosomal stability.
Mr. I. Pinder: Thermography and synovectomy.

Following the meeting a dinner was held at the Priory Hotel.

It is planned to hold the next meeting in Exeter on Friday the 5th May by courtesy of Dr. Harry Hall.

